# PolyMarker: A fast polyploid primer design pipeline

**DOI:** 10.1093/bioinformatics/btv069

**Published:** 2015-02-02

**Authors:** Ricardo H. Ramirez-Gonzalez, Cristobal Uauy, Mario Caccamo

**Affiliations:** ^1^The Genome Analysis Centre (TGAC), Norwich Research Park, Norwich NR4 7UH, UK, ^2^John Innes Centre, Norwich Research Park, Norwich NR4 7UH, UK and ^3^National Institute of Agricultural Botany (NIAB), Cambridge CB3 0LE, UK

## Abstract

**Summary:** The design of genetic markers is of particular relevance in crop breeding programs. Despite many economically important crops being polyploid organisms, the current primer design tools are tailored for diploid species. Bread wheat, for instance, is a hexaploid comprising of three related genomes and the performance of genetic markers is diminished if the primers are not genome specific. PolyMarker is a pipeline that generates SNP markers by selecting candidate primers for a specified genome using local alignments and standard primer design tools to test the viability of the primers. A command line tool and a web interface are available to the community.

**Availability and implementation:** PolyMarker is available as a ruby BioGem: bio-polyploid-tools. Web interface: http://polymarker.tgac.ac.uk.

**Contact:** Ricardo.Ramirez-Gonzalez@tgac.ac.uk

**Supplementary information:** Supplementary data are available at *Bioinformatics* online.

## 1 Introduction

Breeding programs rely on dense genetic maps with markers (e.g. SNPs) that can be used to identify the presence or absence of specific alleles in homozygous or heterozygous state. Standard primer design tools are designed to work in diploids, where genome duplications are rare. Wheat is a polyploid composed of three genomes (A, B and D; referred to as homoeologues) that are related (between 96 and 98% sequence identity), yet distinct. This creates a problem for the design of PCR primers specific to an individual homoeologue. A common approach to circumvent this issue is to manually design primers with a genome specific variant at the 3′ of the primer to increase specificity. We introduce PolyMarker, a tool to automate the design of genome specific primers, thereby reducing the time invested in this process. To make PolyMarker accessible to scientists and breeders, we developed a web server where custom SNPs can be submitted for the design of genome specific assays.

## 2 Description of the tool

First, PolyMarker converts the input marker information (chromosome arm, sequence adjacent to the SNP and parental alleles) into a fasta file with the sequences that can be queried to the genomic reference. The search is performed with Exonerate (Slater and Birney, 2005), with the option –ryo (roll your own format) to facilitate the parsing. The region flanking the SNP, twice the size of the maximum amplification product (200 bp for amplification products up to 100 bp), on the best hit of each chromosome is extracted. A local alignment between homoeologues and paralogues, is refined with MAFFT (Katoh and Standley, 2013), executed using the binder provided in bioruby.

The local alignment is used to produce a mask containing all the variations across homoeologues and the input sequence (Supplementary Fig. S1). The mask indicates the type of variation on each position: (i) *Specific*: homoeologous polymorphism which is only present in the target genome; (ii) *Semi-specific*: homoeologous polymorphism which is found in more than one genome but it discriminates against one of the off-target genomes or when not all the homoeologous sequences were found; (iii) *Non-specific*: when no variation is found across homoeologues; (iv) Homoeologous: The target SNP is present across different chromosomes and; (v) Non-homoeologous: The target SNP is not present across chromosomes. PolyMarker default is to design a three-primer assay for KASP genotyping (LGC Genomics, 2013), that comprises a common primer and two allele-specific primers. Since the allele-specific primers are restricted in position, the common primer is used to incorporate the chromosome specific variants when possible.

To test if the primer candidates are viable Primer3 (Rozen and Skaletsky, 2000) is invoked using the genomic reference of the target chromosome. The starting positions of the primers to distinguish between alleles is selected with the SEQUENCE_FORCE_LEFT_END option of primer3. To design the common primer the option SEQUENCE_FORCE_RIGHT_END is used on two runs of primer3, for the chromosome specific and semi-specific primers. A final run of primer3 is executed without the SEQUENCE_FORCE_RIGHT_END option to find viable primers. After the primers are tested with Primer3, PolyMarker selects a primer pair with the highest specificity.

**Fig. 1.**
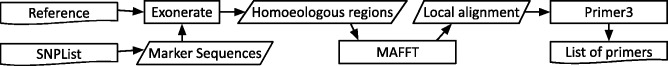
Implementation of PolyMarker. External programs are in squares, trapezoids represent the intermediate files and the document symbols represent inputs and outputs

PolyMarker is a pipeline (Fig. 1) written as a Biogem (Bonnal *et al.*, 2012), extending bioruby (Goto *et al.*, 2010) to extract regions from fasta files and to support operations on nucleotide sequences with IUPAC ambiguity codes (Cornish-Bowden, 1985).

### 2.1 Web interface

Our objective was to make PolyMarker accessible via a web interface where breeders and researchers could submit their markers to custom-design genome-specific SNP assays. A typical output is described in the supplemental material. Batch submission of several markers is possible. The web interface is implemented in java with MySQL database the markers information. The source code of the web interface and the daemon are available for the community to set up a private server.

### 2.2 Example results

The pipeline was developed originally to design KASP assays to validate putative SNPs from RNA-Seq data [28 out of 35 assays polymorphic (80%; Ramirez-Gonzalez *et al.*, 2014)]. PolyMarker was also used to generate KASP assays for the 81 587 markers in the iSelect array from Wang *et al.* (2014) (Supplemental Material).

## 3 Summary

PolyMarker is a pipeline that facilitates the design of primers in polyploid organisms. A web interface is available to design primers for hexaploid wheat. The use of PolyMarker reduces the time spent designing genome specific assays and highlighting homoeologous SNPs.

## Supplementary Material

Supplementary Data
