# Supplementary material for: PolyMarker: A fast polyploid primer design pipeline
Source: Bioinformatics. 2015 Feb 2;31(12):2038–9. doi: 10.1093/bioinformatics/btv069 (PMC4765872; doi:10.1093/bioinformatics/btv069)
Supplement: Supplementary Data [file supp_31_12_2038__index.html]

PolyMarker: A fast polyploid primer design pipeline — PolyMarker: A fast polyploid primer design pipeline — PolyMarker: A fast polyploid primer design pipeline — Supplementary Data 

# PolyMarker: A fast polyploid primer design pipeline

## Supplementary Data

files

**Files in this Data Supplement:**

- Supplementary Data - pdf file
