# Supplementary material for: PolyMarker: A fast polyploid primer design pipeline
Source: Bioinformatics. 2015 Feb 2;31(12):2038–9. doi: 10.1093/bioinformatics/btv069 (PMC4765872; doi:10.1093/bioinformatics/btv069)
Supplement: Supplementary Data [file supp_btv069_SupplementaryMaterial.pdf]

PolyMarker: A fast polyploid primer design pipeline, supplemental material

Ricardo H. Ramirez-Gonzalez, Cristobal Uauy, and Mario Caccamo

December 10, 2014

1 Example results

1.1 Output of PolyMarker in the web interface.

An example output from the web interface of Polymarker is shown in Figure 1. The columns in the output table are: ID, the name in the original input; SNP, the local position of the SNP within the marker in the input; Chr, the target chromosome; CTotal, Total number of contigs; Contig regions, List of contigs where the marker maps; SNP type, Homoeologous if the variation is seen across chromosomes or non-homoeologous if no variation occurs between chromosomes; A and B, The two allele-specific primers with the target SNP in the 3' end; Common, the common primer which incorporates a homoeolgue-specific SNP where possible; Primer type, chromosome specific, chromosome semi-specific or chromosome non-specific; Product size, the size of the PCR product. The mask below the details of each assay includes in the first two rows the sequence of the markers, with dashes (-) when the sequence of the marker is missing but it can be imputed from the alignment to the target chromosome. The following rows contain the local alignment to the chromosomes where the marker maps. The last row is a mask highlighting the features of each base. When a dash (-) is present, no variation occurs between chromosomes and a common primer starting at the given position is labeled as non-specific. Highlighted base (A,C,G or T) indicate positions with a chromosome having a different base to the rest of the chromosomes, primers starting on this positions can be specific or semi-specific, depending on the variation being unique or not to the target chromosome. An ampersand (&) highlights the position of the target SNP when it is non-homoeologous or a colon (:) when it is homoeologous. The designed primers are enclosed by a red outline. a) The SNP (position 151) is non-homoeologus and the common primer (position 152) can be designed as specific to the target B-genome. Positions 121, 124 and 133 are candidate positions for specific primers. Position 130 is a candidate position for a semi-specific primer.

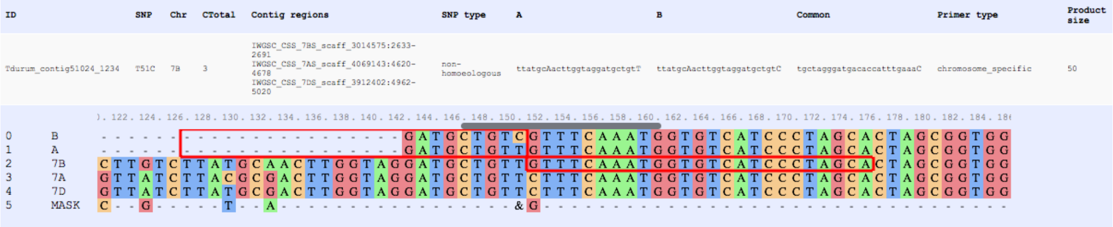

Figure 1: Example output of PolyMarker in the web interface.

Table 1: Count of KASP assays designed for the 40,267 SNP markers located in the genetic map from Wang *et al.* (2014). 4,228 assays did not align to the target chromosome. Not designed: Primer3 could not find viable primers flanking the SNP.

	Homoeologous variant	Varietal SNP	Percentage
Non-specific	1,765	5,857	21.15%
Semi-specific	7,942	6,907	41.20%
Specific	6,813	5,957	35.43%
Not designed	242	556	2.21%
Total	16,762	19,277	36,039

1.2 82k wheat SNPs

We designed primers for KASP assays for the 81,587 markers in the iSelect array from Wang *et al.* (2014) and they are available in the PolyMarker website. We set the target chromosome in 40,267 markers using the published map position (Table 1). For unmapped markers, we assigned a chromosome using the best hit to the flow sorted scaffolds from the International Wheat Genome Sequencing Consortium (2014). 97.5% of the assays where designed and 76% of them are semi-specific or specific, thereby improving their expected performance with respect to randomly designed primers.

References

International Wheat Genome Sequencing Consortium (2014). A chromosome-based draft sequence of the hexaploid bread wheat (*Triticum aestivum*) genome. *Science*, **345**(6194), 1251788–1251788.

Wang, S., Wong, D., Forrest, K., *et al.* (2014). Characterization of polyploid wheat genomic diversity using a high-density 90 000 single nucleotide polymorphism array. *Plant biotechnology journal*, **12**(6), 787–796.
